# Use of CAM among cancer patients: Results of a regional survey in Sweden

**DOI:** 10.1186/s12906-023-03876-2

**Published:** 2023-02-16

**Authors:** Mikael Källman, Stefan Bergström, Tobias Carlsson, Jacob Järås, Georg Holgersson, Johanna Hök Nordberg, Jonas Nilsson, Kathrin Wode, Michael Bergqvist

**Affiliations:** 1 Department of Radiation Sciences, Umeå University, Umeå, Sweden; 2 Centre for Research and Development, Uppsala University/Region Gävleborg, Gävle, Sweden; 3 Department of Oncology, Gävle Hospital, Gävle, Sweden; 4 JRS Statistics AB, Stockholm, Sweden; 5 Department of Oncology, Uppsala University Hospital, Uppsala, Sweden; 6 Regional Cancer Centre Stockholm–Gotland, Stockholm, Sweden; 7 Department of NVS, Karolinska Institution, Stockholm, Sweden; 8 Department of Physiology & Pharmacology, Karolinska Institution, Stockholm, Sweden; 9 Department of Nursing, Umeå University, Umeå, Sweden

**Keywords:** CAM, Cancer, Complementary and alternative medicine, Europe, Oncology, Sweden

## Abstract

**Background:**

The use of complementary and alternative medicine (CAM) by patients is widespread. However, there is a lack of knowledge regarding the extent and details of patient CAM use in Sweden, especially in rural Sweden. The aim of this study was to estimate the extent and characteristics of CAM use among cancer patients in Region Gävleborg.

**Methods:**

A total of 631 questionnaires were distributed to which 376 responses were registered, yielding a response rate of 59.6%. Questionnaires were distributed to oncology patients at their first visit for curative treatment at the Department of Oncology, Gävle Hospital. Palliative patients were recruited at their first visit and during enrollment in palliative outpatient care in their own homes. The characteristics of the respondents were presented with standard descriptive statistics. A multivariable logistic model was fitted to calculate odds ratios (ORs) and identify potential predictors (Age, Gender, Education, Diagnosis) of CAM use post-cancer diagnosis.

**Results:**

54% of all participants reported lifetime CAM use, 34% reported CAM use post-diagnosis. The most common CAM methods used after diagnosis are vitamins, health food preparations, herbal teas, prayer and dietary methods. The most common source of information reported is family and friends. Almost 70% of those who used CAM after their diagnosis stated that they did not discuss their use with healthcare professionals. Most patients reported that they would like some CAM modalities to be offered within conventional care regardless of their own CAM use.

**Conclusions:**

The use of CAM is common among patients with cancer in the region of Gävleborg, and previous studies show a similar use in Sweden in general. Based on the widespread use of CAM and patient interest in discussing CAM use with healthcare professionals, greater attention and focus should be placed on creating a basis for this dialogue. If we, as healthcare professionals, are to emphasise our commitment to providing patient-centred care, we must acknowledge that patients use CAM and are seeking a dialogue about CAM use in their care.

**Supplementary Information:**

The online version contains supplementary material available at 10.1186/s12906-023-03876-2.

## Background

“Complementary medicine” and “alternative medicine” are two terms that describe a group of treatment modalities that are not usually considered part of standard medical treatment. These terms are frequently used interchangeably. However, by definition, alternative medicine is used as an alternative to conventional treatment, whereas complementary treatment is used together with conventional treatment as a supplement. When communicating research, it is common to summarize the two usage patterns under the term “complementary and alternative treatment” (CAM) [1]. CAM treatments can be classified based on how treatment is taken or delivered, which may be nutritional, psychological, physical or a combination of these [2]. Nutritional therapies include substances that can be ingested or injected, such as dietary supplements and herbs [3], whereas psychological and physical treatments include a variety of approaches such as yoga, meditation, acupuncture and massage therapy [4]. There are other approaches that do not fit into any of these groups, such as traditional healers, Ayurvedic medicine and homeopathy [2].

A recent meta-analysis suggests that there has been an increase in CAM use over the last few decades, from about 25% in the 1970s to 49% after 2000 [5]. This is confirmed by another systematic review, which shows a mean prevalence of 51% in the 2000s [6]. In a recent Norwegian study, 79% of patients who currently have or had cancer report the use of CAM [7]. According to a recent cross-sectional study, the use of CAM among Swedish cancer patients is 26% [8]. This is in accordance with the results from a study of 14 European countries, though the results varied significantly from country to country (from 15% CAM use in Greece to 73% in Italy) [9]. The use of CAM seems to be influenced by certain demographic aspects as it has been shown to be relatively more common among young patients, women and patients with high education [6, 8, 10–12].

Previous research contains methodological differences in terms of definitions and categorizations regarding the reasons for CAM use, which makes it difficult to draw conclusions, but according to a systematic review, 38.4% of respondents report a positive experience from CAM use [11, 12]. In a systematic review that included all continents except South America, 74% of respondents stated that the intention to cure and treat cancer was the primary reason for using CAM [6]. In Europe and the Nordic region, the reason for using CAM is mainly to enhance quality of life [7–9]. Other reasons include the feeling of actively doing something, as a way to strengthen the immune system, a measure to counteract the side effects of cancer treatment symptoms and a way to improve the impact of cancer therapy [13]. In a systematic review of the actual effectiveness of CAM in cancer patients, it was shown that some therapies can be effective in alleviating certain symptoms, such as cancer-related fatigue and pain, whereas other therapies show no effect compared to a control group [14, 15]. The American Society of Clinical Oncology (ASCO) has adopted recommendations for the use of complementary therapies such as meditation, yoga and acupuncture to manage the symptoms and side effects of breast cancer treatment [15]. As for the risk of adverse effects, most are described as mild and rapidly resolving [16–19]. In Sweden, a relatively low number of adverse reactions to herbal medicinal products and natural remedies have been spontaneously reported [20, 21]. However, the use of CAM is not entirely safe, as there are direct and indirect risks in some combinations [22, 23]. The direct risks refer, for example, to potentially dangerous interactions between CAM drugs and conventional medicine [24, 25], some of which have led to severe clinical outcomes [26–28]. The indirect risks include, for example, delay or denial of oncological treatment due to communication gaps or lack of knowledge [23]. A lack of knowledge on the part of the healthcare professional is a decisive factor in ruling out dialogue [29].

In a previous study conducted in Sweden, more than half of patients reported spending less than €50 monthly, while 3% spent more than €500 [8].

While CAM use among cancer patients is common, data also show that most patients find it difficult to discuss CAM use with their doctors and nurses [8, 30–32]. Given these discrepancies, there is a need to increase knowledge about CAM use among healthcare providers in order to improve the dialogue with patients.

Previous research has suggested that patients in rural areas have a higher degree of CAM use than patients in urban areas [22, 33]. According to one definition, rural municipalities are municipalities with a population of less than 15,000 residents in the largest urban area [34]. While the reasons behind this different usage pattern are not fully understood, one cause may be a lack of access to conventional care [22, 33]. More research is needed to understand CAM use in rural areas and to create awareness among conventional care providers. Creating an understanding of CAM use in rural healthcare can help change traditional beliefs and practices surrounding health and address some of the key challenges in providing healthcare in rural settings [22]. In Sweden, the use of CAM has mainly been studied in densely populated areas, such as Stockholm [8] and the region of Skåne [25]. Less is known, however, about CAM use in more sparsely populated areas in Sweden, such as Gävleborg County, which has a population of 287,000 inhabitants and a population density of 16 inhabitants per km^2^ [35]. The incidence of new cancer cases per 100,000 people is higher in Gävleborg compared to Sweden as a whole (Men 957/1129 and women 875/941) [36]. The higher incidence rate can at least partly be explained by socio-economic factors, which create a higher susceptibility to certain diseases [37]. The aim of this study was to advance knowledge on this subject by evaluating the use of CAM among cancer patients in Gävleborg county through the following research questions: How common is CAM use? Who uses CAM? Which methods are the most common? what can we learn from healthcare encounters?

## Methods

### Study design

The study uses a cross-sectional design. The questionnaire consisted of 18 questions (with one of the questions prompting 1 or 3 additional follow-up questions depending on the answer) with yes/no, multiple choice and free text answers. The questionnaire was previously developed and used by Molasiottis et al. [9]. Wode et al. translated the questionnaire into Swedish and made some modifications [8]. In collaboration with the co-authors Wode and Hök Nordberg, the questionnaire was slightly modified based on experience from previous surveys. The questionnaire consisted of questions covering topics such as demography, CAM use, reasons for CAM use, methods used and patient experiences with and views regarding CAM (Additional file 1). We used the term post-diagnosis for the use of CAM from the moment of diagnosis to completion of the questionnaire. Data regarding diagnosis came from the participants’ medical records.

Recruitment for the survey started on 13 June 2017, and inclusion ended on December 31, 2018. Written information about the study concept, voluntary participation and confidentiality were included in the survey. The responsible nurse (Study Manager Mikael Källman), who was updated daily about new patients starting chemotherapy via an administrative booking system (ELVIS), informed and asked all new patients if they would agree to inclusion in the study.

The palliative home-care team distributed the questionnaires to patients according to the inclusion and exclusion criteria at the enrolment interview and then informed the study manager.

Two rounds of reminders were sent out to patients who received the survey but did not return a questionnaire or a consent form, the first after 2–4 weeks and the second reminder one month after the first. Before the reminder was sent out, the patient booking system in Region Gävleborg was checked whether or not the patient was deceased.

### Patients

**Fig. 1:**
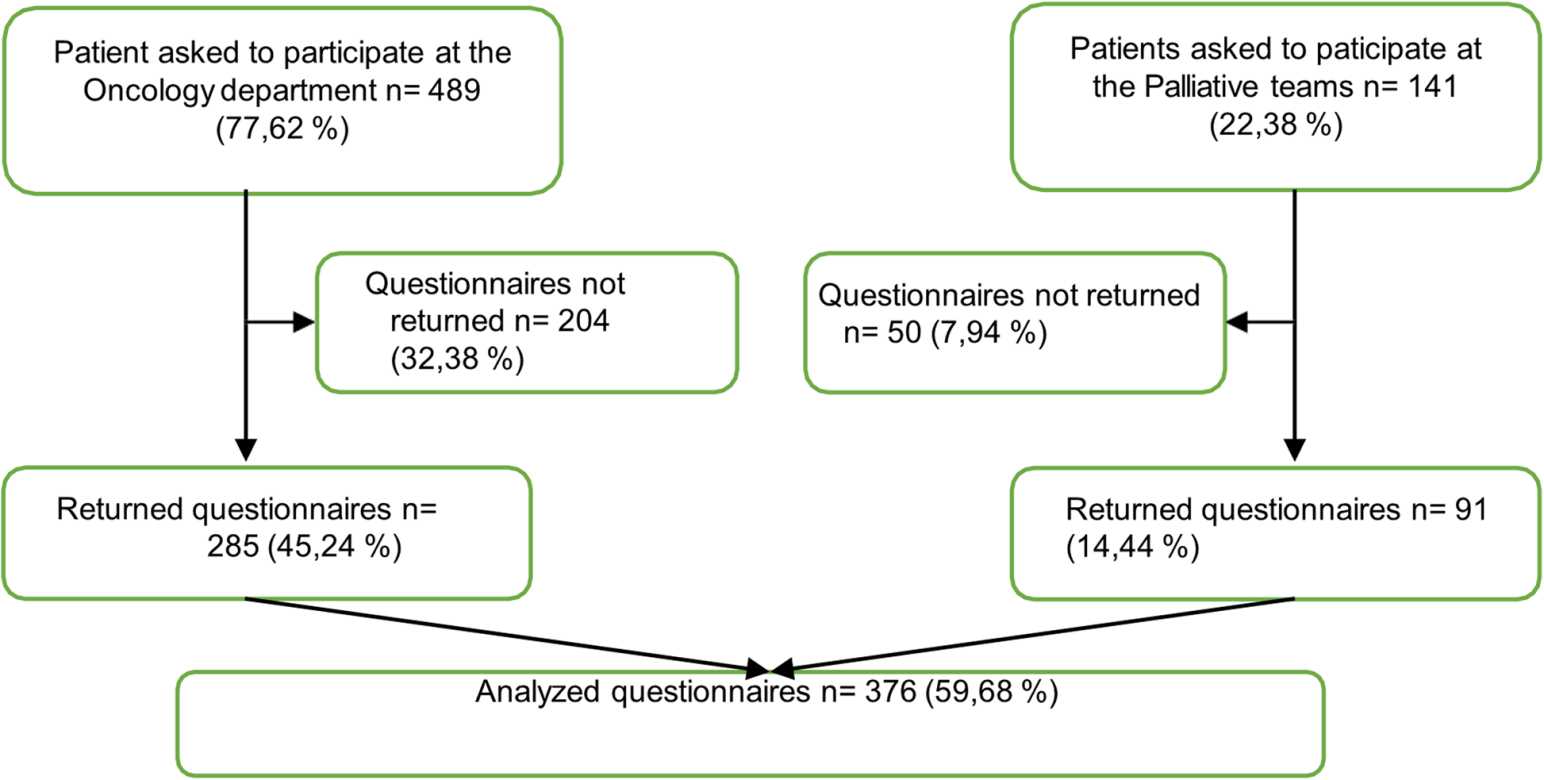
Flow chart of data collection

Patients diagnosed with cancer who started adjuvant chemotherapy treatment and patients enrolled in the palliative home-care team at the oncology clinic, Gävle Hospital, Region Gävleborg, were asked to participate in the study (Fig. 1). To ensure the inclusion of cancer patients throughout the cancer trajectory, we chose to include patients from both curative and palliative settings. Most of the participants resided in Gävleborg County, which consists of the provinces Gästrikland and Hälsingland. Some patients resided in Älvkarleby municipality, which is part of a neighbouring county, but received care at the hospital in Gävleborg due to geographic reasons. Gävle Hospital is by far the largest hospital in the region of Gävleborg and is home to a county oncology clinic; each year, the treatment unit of the oncology department at Gävle Hospital receives approximately 800 to 1,000 unique visits.

Inclusion criteria: Age 18 or above; and starting adjuvant chemotherapy treatment for the first time at the Region Gävleborg Department of Oncology or about to receive best supportive care from the palliative home-care team in Gävle, Region Gävleborg, with or without previous oncological treatment.

Exclusion criteria: Lack of fluency in Swedish, cognitive impairment or other condition that obstructs the ability to understand or fill in a questionnaire as established by the medical assessment of study staff.

### Statistics and data analysis

Quantitative data were presented as median, interquartile range (IQR, 25–75%) and range (minimum–maximum). Categorical data were expressed as proportions.

A multivariable logistic model was fitted to calculate odds ratios (ORs) and identify potential predictors (Age, Gender, Education, Diagnosis) of CAM use post-cancer diagnosis (Table 2). Complete case analysis was applied, which means that only patients with no missing data on the variables of interest were included in the analysis. As a result, two patients were dropped from the analysis as they had missing data on level of education. The final number of patients included in the analysis was 374 patients, 130 of whom had registered CAM usage post diagnosis.

The results were presented as odds ratios (ORs) with corresponding 95% confidence intervals (CIs). *P*-values < 0.05 were considered statistically significant. All statistical calculations were performed using R version 4.2.2 [38].

## Results

### Participant characteristics and CAM use

In this study, we sent questionnaires to a total of 630 patients. Patient characteristics are described in Table 1. A total of 376 patients accepted inclusion in the study (Fig. 1): 285 in the curative segment and 91 in the palliative segment, with a total response rate of 59.68%. The highest rate of non-participation was in the curative arm. The most frequently reported reasons for non-participation in the curative arm were: failed to return survey despite reminders (60%), declined participation due to poor general condition (23%), and deceased (9%). In the palliative arm, the corresponding data were: deceased (76%), and declined participation due to poor general condition (10%). Fifty-four percent of participants reported lifetime CAM use, while 35% of participants reported CAM use after diagnosis.

**Table 1:** Data describing patient characteristics

_Patient characteristics_		Total	CAM = Yes	CAM = No
Age	Median (IQR), min–max	69 (60–75) 23–91	63 (54.5–72.5) 24–90	70 (64–76) 23–91
	NA	0	-	-
Gender	Women	169 (100%)	75 (44.4%)	94 (55.6%)
	Men	207 (100%)	56 (27.1%)	151 (72.9%)
	NA	0	-	-
Education	Elementary school	153 (100%)	31 (20.3%)	122 (79.7%)
	Upper secondary school	126 (100%)	49 (38.9%)	77 (61.1%)
	University or doctoral degree (93/2)	95 (100%)	50 (52.6%)	45 (47.4%)
	NA	2	-	-
Diagnosis	Breast	94 (100%)	44 (46.8%)	50 (53.2%)
	Gastrointestinal	137 (100%)	38 (27.7%)	99 (72.3%)
	Gynaecological	3 (100%)	2 (66.7%)	1 (33.3%)
	Head, neck, lung or skin cancer	39 (100%)	13 (33.3%)	26 (66.7%)
	Haematological	29 (100%)	11 (37.9%)	18 (62.1%)
	Sarcoma	2 (100%)	1 (50%)	1 (50%)
	Urogenital	65 (100%)	20 (30.8%)	45 (69.2%)
	NA	7	-	-

Data from the multivariable logistic regression on the postdiagnosis group (*n* = 130) showed a higher likelihood of CAM use in female patients compared to male patients (OR: 1.94, 95% CI: 1.06–3.57, *p* = 0.032), and a higher rate in patients with a university education compared to patients with only elementary school education (OR: 2.97, 95% CI: 1.60–5.51, *p* < 0.001). Increasing age was associated with lower CAM use (OR: 0.97, 95% CI: 0.95–0.99, *p* = 0.008), while there was no significant association between the type of cancer and use of CAM (Table 2).

**Table 2:** Data describing multivariable logistic regression

Category	n/N, OR (CI 95%)	*p* value
**Age**		
Age	130/374, 0.97 (0.95—0.99)	0.008
**Gender**		
Male	55/205, 1.00 (reference)	
Female	75/169, 1.94 (1.06 – 3.57)	0.032
**Education**		
Elementary school	31/153, 1.00 (reference)	
Upper secondary school	49/126, 1.60 (0.86 – 2.93)	0.134
University	50/95, 2.97 (1.60 – 5.51)	< 0.001
**Diagnosis**		
Breast	44/94, 1.00 (reference)	
Gastrointestinal	38/136, 1.09 (0.53 – 2.24)	0.817
Gynaecological	2/3, 2.85 (0.24 – 34.13)	0.409
Head, neck, lung or skin cancer	13/69, 1.15 (0.46 – 2.88)	0.765
Haematological	11/29, 1.34 (0.51 – 3.53)	0.560
Sarcoma	1/2, 1.67 (0.09 – 29.47)	0.726
Unknown	2/7, 1.20 (0.20 – 7.22)	0.846
Urogenital	19/64, 1.42 (0.56 – 3.58)	0.455

Adjusted for Age, Gender, Education, Diagnosis OR for use of CAM-modality post diagnosis. Numbers in the table represent: No of patients of CAM use, post diagnosis (No=130)/All patients (No=374), OR (95% CI)

The most common CAM methods used prior to diagnosis were massage (11%), vitamins and minerals (10.7%), health-food preparations (10.2%) and acupuncture (10%) (Table 3). Vitamins and minerals (16%), health food preparations (14.7%), herbal teas (6.7%), prayer (6%) and chaga (6%) were the most common post-diagnosis methods used. Most patients (71%) who used CAM after diagnosis used 1–3 different methods or preparations simultaneously (35.9% one method, 19.1% two methods and 1.8% three methods). Ten percent of the patients who used CAM after diagnosis used 7 or more different methods or preparations simultaneously.

**Table 3:** Distribution of selected CAM modalities by categories according to the National Centre for Complementary and Integrative Health

Used CAM modalities	Before^a^ and	after diagnosis % (n)
**Psychological/physical treatments**		
Massage	11% (90)	5.2% (21)
Acupuncture	10% (82)	1.2% (5)
Naprapathy, chiropractic	7.8% (64)	1.5% (6)
Relaxation	6.5% (53)	5.5% (22)
Meditation	3.8% (31)	4.5% (18)
Spiritual guidance, healing	3.8% (31)	3.7% (15)
Yoga	3.8% (31)	4.2% (17)
Prayer	3.7% (30)	6% (24)
Mindfulness	3.2% (26)	2.7% (11)
Zone therapy	2.1% (17)	0.7% (3)
Tai Chi, Qigong	1.7% (14)	0.5% (2)
Support groups	1,1% (9)	1% (4)
Rosen Method Bodywork	0.7% (6)	0.5% (2)
Hypnosis	0.6% (5)	0.2% (1)
Shiatsu	0.6% (5)	- (0)
Art therapy	0.2% (2)	- (0)
**Nutritional products**		
Vitamins, minerals	10.7% (87)	16% (64)
Health food preparations	10.2% (83)	14.7% (59)
Herbal medicines	3.8% (31)	2.7% (11)
Herbal tea	2.6% (21)	6.7% (27)
Aromatherapy	2.1% (17)	0.2% (1)
Colloidal silver	1.1% (9)	3.7% (15)
Injection of mistletoe preparations	- (0)	0.2% (1)
**Other Complementary Health Approaches**		
Homeopathy	2.3% (19)	1.2% (5)
Dietary changes	2% (16)	5.7% (23)
Chaga	1.2% (10)	6% (24)
Birch ash (Potash)	0.9% (7)	1.2% (5)
Energy medicine	0.7% (6)	0.5% (2)
Other modalities	0.5% (4)	2.5% (10)
Ayurveda	0.4% (3)	0.2% (1)
Laser therapy	0.4% (3)	- (0)
Traditional Chinese Medicine	0.4% (3)	0.2% (1)
Anthroposophic medicine	0.1% (1)	0.2% (1)
**Total used modalities**	100%	

^a^All life until diagnosis

The most frequently reported reasons for using CAM in this study were: ‘to improve physical wellbeing 48.9%,’ ‘to improve general wellbeing 45.8%’, and ‘to improve body's ability to fight cancer 38.9%’.

The benefits experienced through the use of CAM are primarily reported as ‘improvement of general wellbeing 39.7%,’ ‘improvement of physical wellbeing 35.9%,’ and ‘improvement of emotional wellbeing 27.5%’.

Eighty-two percent of the patients who used CAM after their diagnosis stated that they paid SEK 500 or less per month for the use of CAM. Seven percent stated that they spent over SEK 1,000 (approx. €100) per month.

Among patients reporting CAM use post-diagnosis (*n* = 131), two patients (1.5%) reported side effects, 106 (80.9%) reported no side effects and 23 (17.6%) provided no response. The reported side effects were nausea, diarrhoea, disturbed sleep due to intake of supplements.

The most common sources of information about CAM were family and friends (40.4%), media (34.2%) and the Internet (22.9%). Respondents reported that they received information from several sources, and 6% stated that they received information from therapists active in complementary and alternative medicine. One-fifth of all respondents stated that they had not heard of CAM before completing the questionnaire.

Only 15% of all respondents stated that they had discussed CAM use with healthcare professionals (Table 4). Of those who used CAM after their diagnosis, 69.8% stated that they did not discuss CAM with healthcare professionals.

**Table 4:** Description of answers from questionnaire regarding the question concerning contact with conventional care

**Have you discussed CAM with health professionals?**						
	Total	%	CAM = Yes (Post diagnosis)	%	CAM = No	%
**Yes**	52	15%	39	30.2%	13	6%
**No**	294	85%	90	69.8%	204	94%
**Missing**	30	-	2	-	28	-
**Should providers be able to inform?**						
	Total	%	CAM = Yes (Post diagnosis)	%	CAM = No	%
**Yes**	194	53.9%	90	69.8%	104	45%
**No**	16	4.4%	3	2.3%	13	5.6%
**No opinion**	150	41.7%	36	27.9%	114	49.4%
**Missing**	16	-	2	-	14	-
**Should CAM be offered within conventional care?**						
	Total	%	CAM = Yes (Post diagnosis)	%	CAM = No	%
**Yes**	224	68.5%	97	80.2%	127	61.7%
**No**	103	31.5%	24	19.8%	79	38.3%
**Missing**	49	-	10	-	39	-

Over fifty percent of study participants had the opinion that conventional care should provide information about CAM, around 40% of respondents did not take a position on the issue and only approximately 5% stated that conventional care should *not* provide information about CAM. Among CAM users, more than 65% stated that healthcare professionals should be able to provide information about CAM, while the corresponding number among non-users was 45%.

A majority of the patients included in the study state that some CAM should be offered in the context of conventional care, while 80% of patients who used CAM after diagnosis and 61% of patients who did not use CAM stated that CAM should be offered in conventional care (Table 4).

## Discussion

In this paper, more than half (54%) of participants reported lifetime CAM use. This is higher than the 34% previously reported in Swedish cancer patients [8]. Given recall bias, estimating "lifetime CAM use" can be assumed to be more difficult than estimating "CAM use post-diagnosis". The first period can extend over 50–60 years, while CAM use after cancer diagnosis is a more definitive period of time. In this study, 35% of participants reported CAM use post-diagnosis in contrast to 26% in a previous Swedish study [8]. The findings here suggest more extensive use of CAM in the less densely populated area of Gävleborg compared to the Stockholm region, which is in line with previous studies which have suggested a greater use of CAM in sparsely populated regions [22, 33]. Another aspect that may have influenced these higher usage numbers is, that our data even include palliative patients without any oncological treatment in contrast to the study from Stockholm. However, due to differences in study design between different studies about CAM use, it is difficult to draw definite conclusions [8, 22, 39]. The study had a response rate of 58% in the adjuvant part and 64% in the palliative part.

In accordance with previous reports, the multivariable logistic model indicated that the probability of using CAM was higher in younger patients, in female patients and in patients with a university education [8, 9, 40, 41]. Type of cancer, on the other hand, showed no significant association with the use of CAM, which is in accordance with some previous studies [6, 42]. However, there are also studies showing different frequencies of CAM use among different cancer diagnoses [43].

Our data suggests that due to the reported lack of dialogue regarding CAM, there is a discrepancy between patients’ desire to receive information from healthcare professionals and the information they actually receive. According to this study, at least one third of patients use CAM, and a majority of these patients report a lack of dialogue about CAM with healthcare professionals. It is also important to note that while 15% of total respondents discussed CAM use with healthcare professionals, 30% of CAM users had such discussions. These findings are similar to previous studies [8] that indicate that dialogue is still patient-driven. In this cohort, the most common sources of information about CAM were family, media and the Internet, which is in line with previous reports [8, 44, 45] and further reinforces the need for quality-assured information. The lack of CAM knowledge among Swedish healthcare professionals has been reported previously [46], and a Swedish CAM investigation carried out on behalf of the government found the same conclusion [47]. Furthermore, a majority of patients (53.9%) state that healthcare providers should be able to inform patients about CAM use. There is thus a need and desire for patients to receive information regarding CAM use. Furthermore, it is important that the care profession is not dismissive of patient CAM use [48]. Discussion of CAM use should be based on the patient's opinions, professional experience and evidence [3].

Since 85% of all respondents reported that they had no discussion with healthcare providers regarding CAM use, and since we know that some herbs can affect drug uptake (e.g. Echinacea may reduce the effects of immunosuppressants) [40], these findings suggest a significant potential risk of interactions between CAM interventions and cancer treatments. In our study, Chaga was a common modality used by patients. Another study found that there is a potential theoretical risk of interaction, but risks are still unexplored in humans. The clinical relevance of the potential interaction is therefore uncertain [49]. We recommend that this should be documented and further research should be done in this area. In line with previous reports [8], CAM users in this cohort were more likely to have discussed CAM with healthcare providers compared to non-users (30% vs 6%). This points a concern that has been raised in previous discussions [8], whether poorly informed physicians, with a negative mindset about CAM, may induce patient anxiety and lead patients to abstain from discussing CAM use with healthcare providers [50].

The most common reasons for CAM use match the reported benefits: increased physical and emotional wellbeing. Other common reasons, including ‘to improve body’s ability to fight cancer’ and ‘to fight cancer’ (9.9% and 26%, respectively), did not match as well with their perceived benefits. However, a majority of patients (68.5%) stated that some CAM modalities should be offered within conventional cancer care, which is in line with the study by Wode et al. [8].

We also report that patients use a variety of CAM modalities, often in combination, which is also in line with previous studies [8, 48]. Our findings regarding the number of simultaneous modalities have not been analysed in depth, but can be seen as slightly higher than the findings in previous studies [51]. Most interestingly, the type of CAM used before and after a cancer diagnosis seems to vary. The use of massage and acupuncture decreased, while the use of vitamins and minerals, health-food preparations, Chaga and herbal teas increased. These results are consistent with previous studies [48, 52].

Since one of the most commonly cited reasons for CAM use was to improve wellbeing and the body’s ability to fight cancer, patients may believe that dietary changes and food supplements can accomplish these goals. However, the inability to link the user's motives to individual methods is of course a weakness of the present study. The relatively high pre-diagnosis use of massage does not factor in when these methods were actually tried, making this result ambiguous.

Only two CAM users (1.5%) who responded to the survey reported side effects. The reported adverse effects are lower than those previously described in studies 4.4% and 5.6% [8, 42]. In all, 106 (80.9%) patients reported no side effects, and 23 (17.6%) patients provided no response.

One limitation of the study is the selection of patients, which come exclusively from a small region in Sweden and may not be representative of the country as a whole. The total response rate of 59% may be seen as a limitation of this study but is consistent with response rates to previous studies [5].

We did not see any clear signs of self-selection bias (i.e. that CAM users would be willing to participate to a higher degree than non-users), as the age and gender distribution of those who could not respond, or chose not to respond, were analysed and yielded distributions that are very similar to that of the respondent group. While this does not preclude sample bias, the similarity in age and gender distribution between the two groups make it less likely.

## Conclusions

In the present study, more than half of the cancer patients in the Swedish region of Gävleborg reported lifetime CAM use, and about one third reported CAM use post-diagnosis. We also found that almost 70% of those who used CAM after their diagnosis stated that they did not discuss this with healthcare professionals, while about two thirds of these patients wanted their healthcare providers to be able to provide information about CAM. Based on the widespread use of CAM among patients and the desire for dialogue with healthcare professionals, greater attention and focus should be placed on creating a basis for this dialogue. If we, as healthcare professionals, are to emphasise our commitment to providing patient-centred care, we must acknowledge that patients use CAM and are seeking a dialogue about CAM use in their care.

## Supplementary Information

**Additional file 1.**

## Data Availability

Due to privacy regulations, the data generated and analysed is not available to the public. An adapted version is available from the corresponding author upon reasonable request.

## Abbreviation

CAM: Complementary and alternative medicine
