# Supplementary material for: Use of CAM among cancer patients: Results of a regional survey in Sweden
Source: BMC Complement Med Ther. 2023 Feb 16;23:51. doi: 10.1186/s12906-023-03876-2 (PMC9933304; doi:10.1186/s12906-023-03876-2)
Supplement: Supplementary file 1 — Additional file 1. [file 12906_2023_3876_MOESM1_ESM.pdf]

Region Gävleborg
REGIONALT CANCERCENTRUM STOCKHOLM GOTLAND

1. Age: ____

2. Are you:

☐	Man
☐	Woman

3. What is the highest educational level you have completed?

☐	Elementary school
☐	High school
☐	College/university
☐	Post-graduate education

4. Have you at any point in life used any form of so called complementary and alternative methods?

☐ Yes Continue with question 6

☐ No Continue with question 5

5. Please check the main reason(s) for not using any complementary and alternative methods:

You can check several answer options.

☐	Never thought about using complementary and alternative methods
☐	I do not believe in methods lacking scientific proof
☐	I am happy with the care and treatment I receive at the department of oncology
☐	I cannot afford complementary and/or alternative methods
☐	I´ve thought about using complementary and alternative methods but was discourag[illegible] by family/friends/health professionals and decided therefore not to do so
☐	Other reason: ____ ____ ____ ____ ____

6. Have you used any of the following complementary and alternative methods before or after your cancer diagnosis? What of these methods are you currently using?
You can check several answer options

	**Used before cancer diagnosis**	**Used after cancer diagnosis**
Acupuncture	☐	☐
Spiritual guidance, healing	☐	☐
Anthroposofic medicine	☐	☐
Aroma therapy	☐	☐
Relaxation	☐	☐
Ayurveda	☐	☐
Birch ash	☐	☐
Prayer	☐	☐
Energy medicine	☐	☐
Homeopathy	☐	☐
Hypnosis	☐	☐
Collodialt silver	☐	☐
Art therapy	☐	☐
Changes in diet beyond those recommended at the department of oncology	☐	☐
Laser theraphy	☐	☐
Massage	☐	☐
Mindfulness	☐	☐
Meditation	☐	☐
Mistletoe injections	☐	☐
Naprapathy, Chiropractic treatment	☐	☐
Natural preparations / health food preparations, Ex. omega-3, turmeric, apricot kernels, aloe vera, russian root, dandelion root<br>*Please specify:*	☐	☐
___________________	☐	☐
___________________	☐	☐
___________________	☐	☐
___________________	☐	☐

________________	☐	☐
Herbal Remedies /<br>Herbal<br>medicines from pharmacies, ex. echinacea, garlic<br>Please specify:	☐	☐
________________	☐	☐
________________	☐	☐
________________	☐	☐
________________	☐	☐
Rosen theraphy	☐	☐
Shiatsu	☐	☐
Support groups	☐	☐
Tai Qi/Qi gong	☐	☐
Herbal tea (e.g. Green tea, Pau D'arco)<br>Please specify:	☐	☐
________________	☐	☐
________________	☐	☐
Tea from ticks (e.g. Chaga)<br>Please specify:	☐	☐
________________	☐	☐
________________	☐	☐
Traditional Chinese Medicine	☐	☐
Vitamins and minerals, e.g. C-vitamin, D-vitamin, multivitamins, selen<br>*:*	☐	☐
________________	☐	☐
________________	☐	☐
________________	☐	☐
________________	☐	☐
________________	☐	☐
Yoga	☐	☐
Reflexology	☐	☐
**Others, please specify:**	☐	☐
________________	☐	☐
________________	☐	☐

If you have not used any of the methods above after your cancer diagnosis→Continue with question 15.

7. If you began using complementary and/or alternative therapies after you knew you had cancer, when did you begin using these therapies?

____
____
____

8. Please give the reason why you are using or have used complementary and alternative methods.

You can check several answer options. Specify the method in the right column.

		Method
☐	To fight cancer	
☐	To improve the body´s ability to fight cancer	
☐	To improve physical well-being	
☐	To improve emotional well-being	
☐	To improve general well-being	
☐	To relieve symptoms from cancer disease	
☐	To relieve side-effects from oncological treatment	
☐	Other:____	

9. What <u>benefit</u> have you experienced form the used complementary and alternative methods?

You can check several answer options. Specify the method in the right column.

		Method
☐	Direct decrease of tumor as a result from using complementary and alternative methods	
☐	Increase in my body´s ability to fight cancer	
☐	Improvement of physical well-being	
☐	Improvement of emotional well-being	
☐	To improve general well-being	
☐	Reduction of symptoms from cancer disease	
☐	Reduction of side-effects from oncological treatment	
☐	Other:____	
☐	No benefit at all	

10. Have you experienced any ill effects or side-effects, which you attribute to the complementary and alternative therapy?

☐ Yes ➔ *Specify **what** and I relation to what **method** below*

☐ No

Method	Ill effect or side-effect

11. How much do you currently spend on complementary and alternative methods (incl. products and treatments) in one month? (Please check)

☐	0 SEK
☐	500 SEK or less
☐	501-1000 SEK
☐	1001-5000 SEK
☐	5001-10 000 SEK
☐	10 000 SEK or more

12. Do you think the complementary and alternative methods you have used were worth the money?

☐ Yes

☐ No

Please comment:

___

___

___

.

13. How satisfied are you with the complementary and alternative method(s) you have used since your cancer diagnosis?
Mark a number 1-4.

1	2	3	4
☐	☐	☐	☐
Not satisfied at all	*A bit satisfied*	*Quite satisfied*	*Very satisfied*

Please comment:

____
____
____

14. Have you discussed use of complementary and alternative methods with your doctor or nurse at the department of oncology/palliative team?

☐ Yes ➔ *Continue with question 14a -14c.*

☐ No ➔ *Continue with question 14d.*

14a. Did you talk to a doctor, nurse or other professional?

☐ Nurse

☐ Doctor

☐ Other professional___________________________

14b. If so, how was the conversation?

____
____
____
____

14c. If yes, were you satisfied with the conversation?

☐ Yes

☐ No

Please comment:

____
____
____
____

14d. If no, please state the reason

____
____
____
____

15. What was your source of information about complementary and alternative methods?

You can check several answer options.

☐	No one, never heard of it until today
☐	The media (TV, magazines, newspapers)
☐	Internet
☐	Family and friends
☐	Practitioners of complementary and alternative therapies
☐	From primary care/department of oncology/home care
☐	Other:____

16. In your opinion, should health care providers at the oncology department/palliative team be able to answer questions related to complementary and alternative methods??

☐ Yes

☐ No

☐ No opinion

Region Gävleborg

REGIONALT CANCERCENTRUM STOCKHOLM GOTLAND

.

17. How would you prefer to receive information about complementary and alternative methods from the oncology department/palliative team?

☐	Written information (webpage or brochure)
☐	E-mail/chat forum with possibility to ask questions
☐	Personal meeting with a competent person
☐	Lecture
☐	Other: ____

18. In your opinion should certain complementary and alternative methods be offered within oncological care/palliative team?

☐ Yes

☐ No

Please comment:

____

____

Thank you for your participation!
